# Loss of Pluripotency in Human Embryonic Stem Cells Directly Correlates with an Increase in Nuclear Zinc

**DOI:** 10.1371/journal.pone.0012308

**Published:** 2010-08-20

**Authors:** Janet L. Wolford, Yasmin Chishti, Qiaoling Jin, Jesse Ward, Liaohai Chen, Stefan Vogt, Lydia Finney

**Affiliations:** 1 Biosciences Division, Advanced Photon Source, Argonne National Laboratory, Argonne, Illinois, United States of America; 2 X-ray Science Division, Advanced Photon Source, Argonne National Laboratory, Argonne, Illinois, United States of America; Wellcome Trust Centre for Stem Cell Research, United Kingdom

## Abstract

The pluripotency of human embryonic stem cells (hESCs) is important to investigations of early development and to cell replacement therapy, but the mechanism behind pluripotency is incompletely understood. Zinc has been shown to play a key role in differentiation of non-pluripotent cell types, but here its role in hESCs is directly examined. By mapping the distribution of metals in hESCs at high resolution by x-ray fluorescence microprobe (XFM) and by analyzing subcellular metal content, we have found evidence that loss of pluripotency is directly correlated with an increase in nuclear zinc. Zinc elevation not only redefines our understanding of the mechanisms that support pluripotency, but also may act as a biomarker and an intervention point for stem cell differentiation.

## Introduction

Human embryonic stem cells (hESCs) are defined by two characteristics: unlimited power of self- renewal and pluripotency, which is the ability to become any human cell type. These features make hESCs the ideal system for the study of pluripotency maintenance and early differentiation, which is currently defined as a balancing act between three forces. First, a set of proteins, especially Sox2, Oct4 and Nanog, maintain pluripotency. In opposition, there are many key regulatory factors that promote differentiation and eradicate pluripotency [1]. Thirdly, chromatin remodeling, i.e. the altering of DNA accessibility by either histone modification or changes in nucleosomes and higher order structures, has also proven to play a significant role in hESC differentiation [2], [3]. Although zinc has been recognized as a player in the differentiation and proliferation of other cell types [4], its role in hESC pluripotency and early differentiation has not been addressed by studies prior to this work.

Zinc is primarily recognized in biology as a structural protein folding element found in zinc finger motifs, however it has many other cellular functions. While proteins with zinc fingers are key players in protein repression, zinc is a structural element of many other proteins, and can even play a catalytic or coactive role [5], [6]. Like other biologically relevant transition metals, it is rarely found in an uncoordinated state [7]; and in eukaryotic cells, zinc is typically chaperoned by the small, cytosolic, metal-chelating protein, metallothionein [8], [9]. But in specialized cells, such as glutamatergic neurons [10] and mammary epithelial cells [11], vesicular pools of free zinc exist. Studies identifying these pools, and others like them, utilize fluorescent zinc probes, small fluorescent molecules that are both sensitive and specific for zinc such as zinquin [12], FluoZin-3 [13], Zinpyr-1 [14], and ZnAF-1F [15], [16]. Because the affinity of such fluorophores is typically less than that of most proteins, they are considered “free” zinc indicators, but cannot allude to total zinc content. Zinc can also play a role in signal transduction, modulating the activity of cyclic nucleotide phophodiesterase, mitogen-activated protein kinase, protein kinase C, and several other messengers in signaling cascades [4]. Therefore, zinc is virtually as critical to cellular function as calcium, although far less is understood with regard to its homeostasis.

Studies have shown that in a few specialized cell types, differentiation and proliferation appears to correlate with zinc concentration and subcellular localization. In myoblast cultures, zinc chelation blocks the increase of creatinine kinase mRNA levels, a crucial step for myoblast differentiation [17]. In 3T3-L1 preadipocytes, differentiation begins with proliferation, a step associated with elevated cellular zinc and nuclear translocation of metallothionein. After differentiation, zinc content drops and metallothionein is again cytosolic [18]. In these studies, the subcellular concentration and location of zinc, however, are less clear. In a more direct approach to subcellular zinc analysis, one study found that nuclear zinc content increases as HL-60 myeloid leukemia cells differentiate into macrophages [19]. Clearly, a role for zinc in non-pluripotent cellular differentiation exists, however no unifying mechanism has been uncovered.

Given zinc's many intracellular capabilities, we hypothesized that changes in zinc's subcellular localization occur during differentiation in hESCs. To test this hypothesis, we directly imaged zinc in both pluripotent and differentiating hESCs. Subcellular tracking and quantification of metals was achieved through use of the x-ray fluorescence microprobe (XFM) of beam-line 2-ID-E of the Advanced Photon Source. Cells were maintained and differentiated in monolayer colonies, as opposed to embryoid body differentiation methods, to facilitate imaging. XFM analysis with submicron resolution has detailed both exogenous and endogenous metals in biological samples [20]–[22]. Although chelation studies have been helpful in defining a role for zinc in many biological processes, we have found that hESCs are highly sensitive and do not survive even very mild chelation conditions (data not shown). By comparing XFM analysis with imaging using the fluorescent zinc probe, FluoZin-3, we discovered that nuclear zinc concentrations do increase with loss of pluripotency, independent of the differentiation methods used in this work, and that this nuclear zinc does not appear accessible to fluorescent probes. Based on all of this, we propose that the zinc involved in cellular differentiation is protein or small molecule bound.

## Results

### Spontaneous hESC differentiation

**Figure 1:**
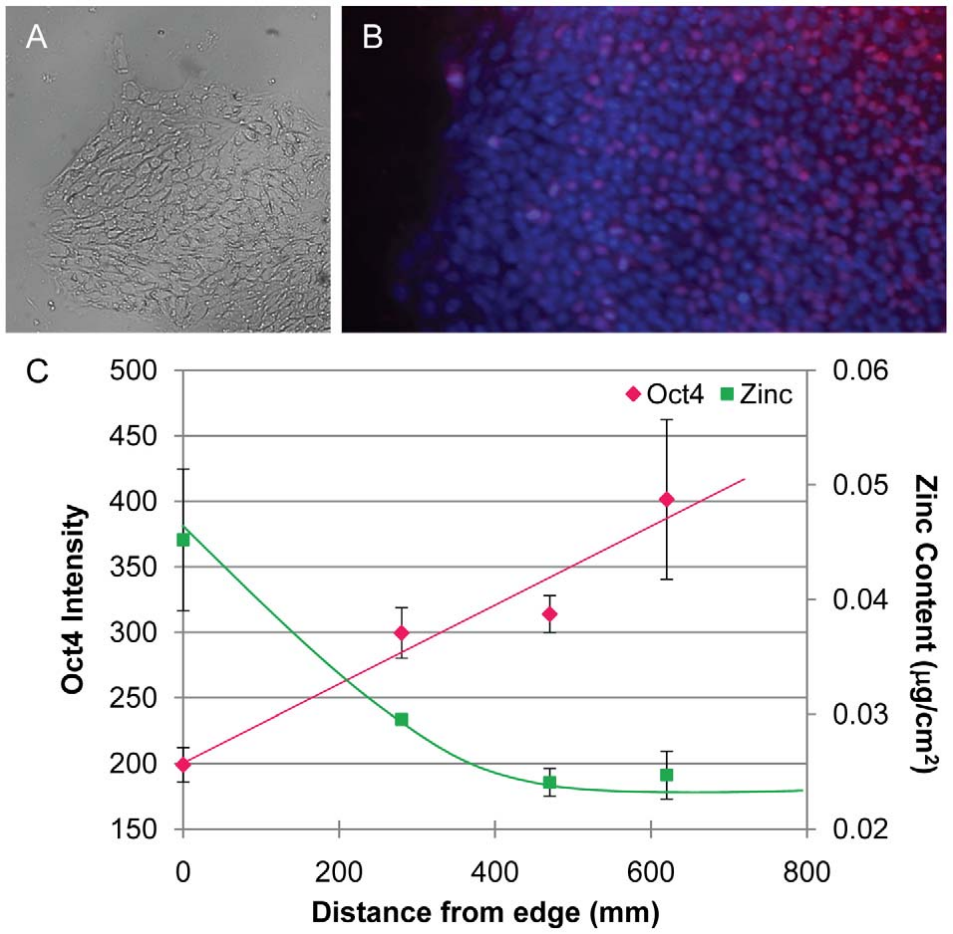
Spontaneous differentiation correlates with increased nuclear zinc content at hESC colony borders. A. DIC (10x) image of hESC colony showing a change in morphology at colony border. B. Oct4 labeling lost at colony border but maintained in center of colony indicates that pluripotency is maintained centrally. C. Graph depicting an inverse relationship between quantified Oct4 signal and zinc content from XFM analysis across the colony. Results are plotted as mean ± SEM.

To begin exploring the role of zinc in hESCs, we examined H9 cells in standard growing conditions. After 7 days, hESCs under standard growth conditions begin to spontaneously differentiate along colony borders as observed in DIC images (Figure 1A) and as detected by the loss of Oct4 immunofluorescence (Figure 1B), a marker of pluripotency [23]. A measure of pluripotency across the colony was made by averaging the Oct4 signal from cells in a single field of view and comparing it to the average signal from adjacent fields in the colony. This analysis demonstrated a gradual increase in pluripotency from the colony border to the colony center rather than a sudden loss of pluripotency at the border (Figure 1C). Metal distributions (P to Zn on the atomic chart by K-line fluorescence) throughout these colonies were then examined at the Advanced Photon Source by XFM analysis. This analysis demonstrated cellular elemental distributions common to other cell types with the majority of P and Zn found in the nucleus. Colonies were essentially imaged by raster scanning areas of 5–20 cells at a time with the XFM and then repeating the imaging on congruous areas across the colony diameter. With this method, we found that the Zn fluorescence intensity in the nucleus was dependent on relative location of the cell in the colony, i.e. average nuclear zinc content was higher at the edge of the colony and lower toward the center. By comparing this data with that of the Oct4 imaging, we found an inverse relationship between pluripotency and zinc content (Figure 1C).

### Retinoic acid treatment

**Figure 2:**
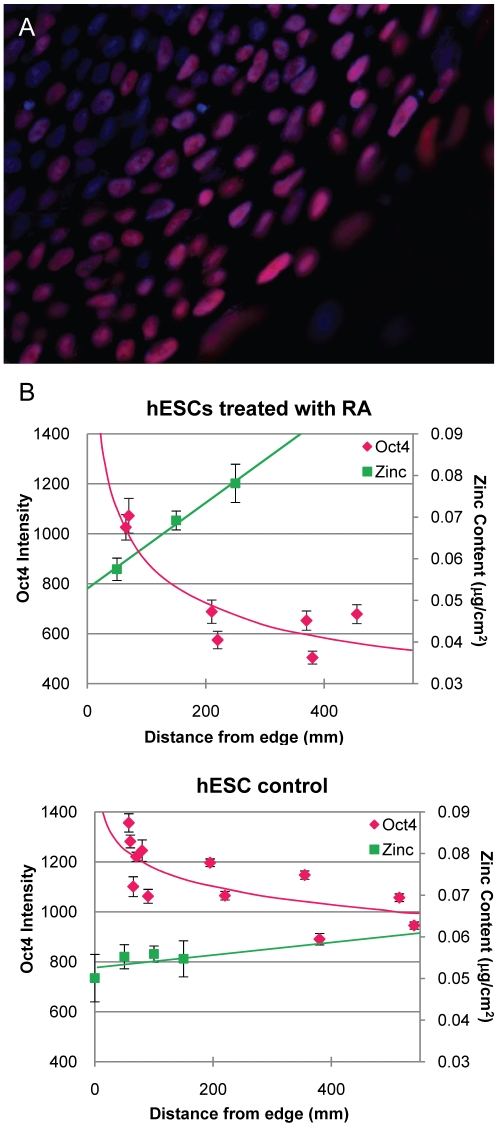
RA induced differentiation correlates with increased nuclear zinc content at the center of hESC colonies. A. Oct4 (pink) lost at center of hESC colonies treated with RA. Cells are counterstained with Hoechst (blue). B. Graphs depict quantified Oct4 intensity vs. total zinc content from XRF of control (i) and RA treated (ii) hESCs across a typical colony. Results are plotted as mean ± SEM.

To further explore the relationship between differentiation and zinc content, the process of differentiation was perturbed by the presence of 10 µM of retinoic acid (RA), the action of which is pleiotropic in hESC differentiation [24]. After five days of RA exposure without the addition of any other growth factors, we observed a morphological change and loss of pluripotency as demonstrated by Oct4 staining (Figure 2A), but in an inverse pattern to spontaneous differentiation: loss of pluripotency occurred at the center of the colony as opposed to the edge (Figure 2B). No cells demonstrated positive Sox-1 [25] nor smooth muscle α-actin [26], suggesting that, with RA alone, neither a neuroepithelial nor a smooth muscle phenotype had been achieved (data not shown). With RA treatment, XFM analysis demonstrated higher average zinc content in nuclei at the center of the colony and lower in nuclei near the edge. The sizes of cell nuclei were the same at the edges as in the center of the colony (within 1 SEM); therefore, we also demonstrated higher total nuclear zinc content in cells at the center of the colony. These results were consistent with those from spontaneous differentiation: in colony regions where Oct4 levels were high, zinc content was low and vice versa (Figure 2B). Thus, we demonstrated that the elevated zinc content was related to loss of pluripotency independent of the position of a cell in the colony.

### Activin A directed differentiation to endoderm

**Figure 3:**
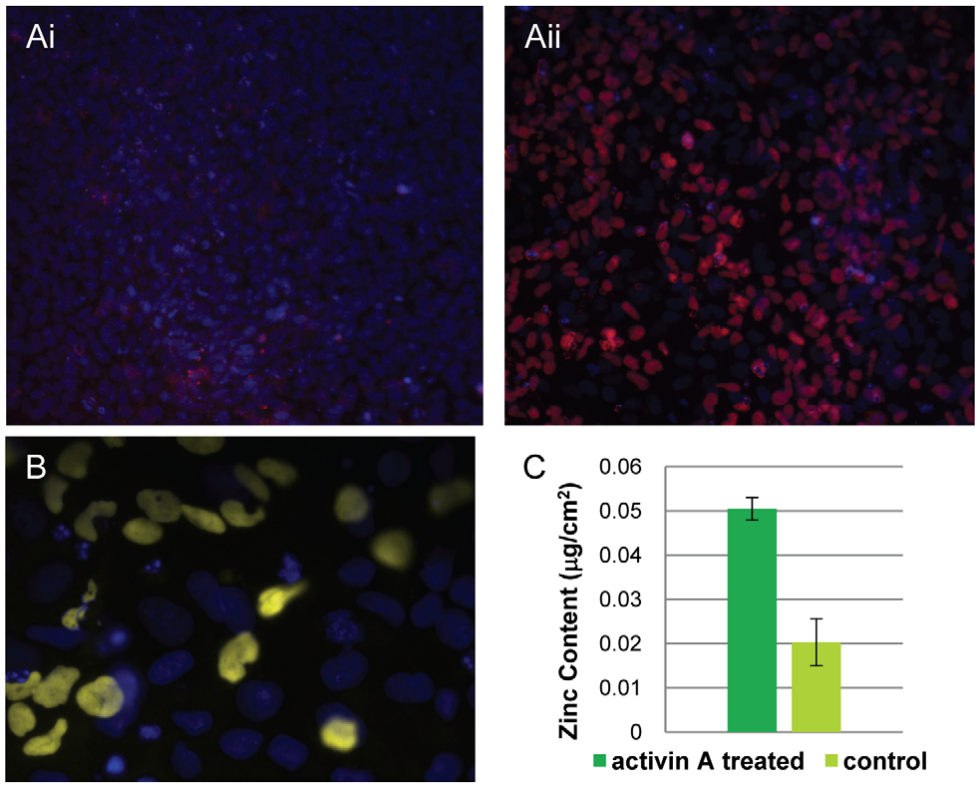
Activin A induced differentiated hESCs have increased zinc content. A. Control (i) and Activin A treated (ii) hESCs stained with Oct 4 (pink) and counterstained with Hoechst (blue). B. Sox17 expression (yellow) in Activin A treated cells. Cells are counterstained with Hoechst (blue). C. Graph depicting zinc content of Activin A treated cells vs. control. Results are graphed as mean ± SEM.

After observing that zinc content varied with loss of pluripotency across a colony, we examined hESCs differentiated into endoderm by activin A induction [27], [28]. After exposing hESCs to 100 ng/mL of activin A from post-passaging day 4 to 7, we detected a loss of Oct4 in approximately 50% of the cells (Figure 3A). We also detected Sox17 by immunofluorescence in roughly 50% in the cells, confirming the presence of endoderm (Figure 3B). Differentiation was independent of cell position in the colony. XFM analysis demonstrated a statistically significant difference in zinc content between the activin A treated and control hESCs, with activin A treated cells being approximately 2.5 times higher in nuclear zinc content (Figure 3C). Although the total zinc content in the control cells and the activin A treated cells was lower than that of pluripotent and early differentiated cells, respectively, from the earlier experiments, this was expected due to the reduced serum content in the media for activin A experiments [29].

### FluoZin-3 staining

**Figure 4:**
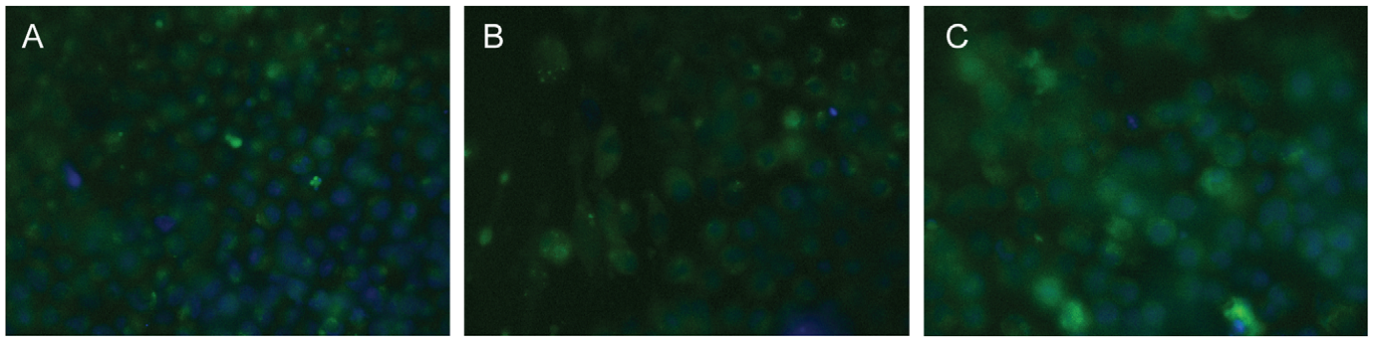
FluoZin-3 staining is unchanged with induction of differentiation. FluoZin-3 is green, Hoechst 33342 is blue. A. Control H9 cells. B. RA treated cells. C. Activin A treated cells. All images are representative of roughly 20 fields of view from three experimental replicates.

In our XFM analysis, the zinc content of the cytosol was largely unexplored due to the large nucleus to cytosol ratio in hESCs, the difficulty of accurately defining borders of cells grown in monolayer colonies, and the low cytosolic zinc content when compared to the nucleus. Given that the nuclear zinc content of a hESC increases with differentiation, the question arises: what is the source of this zinc? Although there is great debate regarding the presence of free zinc pools in non-specialized cells, we hypothesized that hESCs may have such a store that changes with loss of pluripotency. Unlike XFM analysis, which measures total zinc content, fluorescent zinc-specific probes, like FluoZin-3 [13], [30], imaged with light microscopy have been used to illuminate the “free” zinc state. The hESC cells, including controls, RA treated, and activin A treated hESCs, were stained with FluoZin-3 and Hoechst. Diffuse faint cytoplasmic staining with few bright speckles was observed in the cytoplasm of cells from each treatment (Figure 4). These bright speckles may be either vesicular zinc pools, autofluorescence, or dye precipitation. Although the significance of the speckled staining is undetermined and may be artifactual, the three treatments all demonstrate the same staining pattern. Therefore, the cytoplasmic zinc pool that is available to this probe appears to be unchanged with loss of pluripotency. In no treatment was fluorescence seen in the nucleus which is observed to have the highest zinc content by XFM analysis. FluoZin-3 is known to enter the nucleus [31], therefore the lack of nuclear fluorescence suggests that nuclear zinc is bound to another molecule making it neither thermodynamically nor kinetically available to the fluorescent probe.

### Mitotic chromosomes

**Figure 5:**
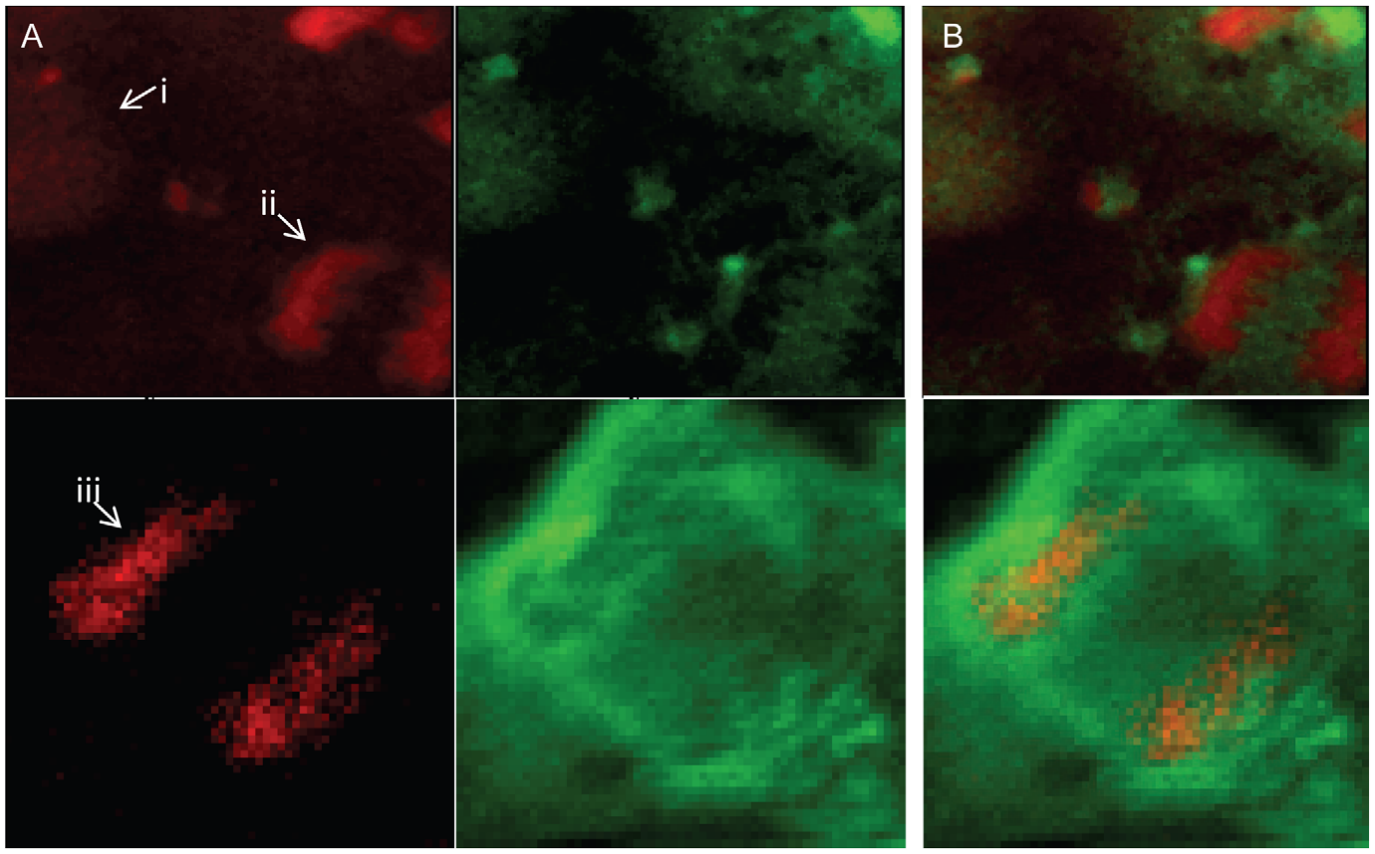
Zinc excluded from condensed chromosomes of mitotic cell. A. Raster scans mapping Kα line fluorescence of phosphorus (red) and zinc (green) of an interphase (i) and mitotic (ii, iii) cells. B. Overlay demonstrating overlap of zinc and phosphorus in the interphase cell and mutual exclusion in the anaphase cells. The first row of images was obtained with x-ray beam spot size of 0.4×0.3 um with step sizes of 0.2 um. Second row was obtained with 0.6×0.8 um spot size and a 0.5 um step size.

In both pluripotent and differentiated cells, we have observed zinc concentrated in the nucleus, diffusely across the entire nucleus. For a closer examination of cell nuclei, the x-ray beam was focused to a smaller spot size and finer resolution raster scanning was performed. Although heterochromatin and euchromatin were not discrete enough to examine separately with XFM, mitotic cells in anaphase (as determined by Hoechst staining) shed some light on the zinc content of condensed chromatin. We observed the x-ray fluorescent phosphorus signal from the DNA backbone as predicted by the Hoechst staining: diffuse throughout the nucleus in the majority of cells, condensed and migrating to poles in the mitotic cells (Figure 5A). The x-ray zinc fluorescence was also diffuse throughout the nuclei of the majority of cells; but in mitotic cells, it was excluded from the portion of the nucleus with high phosphorus signal (Figure 5B). These results seem to question the role of zinc in maintaining condensed chromatin structure, at the very least painting a more complex picture than may have been previously envisioned for this function of zinc.

## Discussion

In this work, we demonstrated that under three differentiation conditions, nuclear zinc content of hESCs increased in the early stages of differentiation. This zinc increase was independent of colony position and remained elevated at least to the point of differentiation to endoderm. Given the low cytosolic to nuclear zinc ratio and unchanging cytosolic FluoZin-3 staining, we postulate that the increase in bound nuclear zinc results from import of extracellular zinc as opposed to mobilization of cytosolic sources. The nuclear zinc elevation does not appear to be an influx of “free” zinc, but the coordinating molecule is as of yet unknown. We are therefore left with three broad possibilities for zinc coordinating molecules: DNA, zinc containing proteins, or small molecules.

In this work, we considered that the increase in nuclear zinc may bind directly to DNA or to DNA folding proteins, perhaps as a prerequisite for chromatin folding and inactivation as heterochromatin increases in the differentiating ESC [3]. This hypothesis arises partly from reports that RA can induce chromatin remodeling [32], [33] and from the idea that nucleosomes have a potential zinc binding site [34], [35]. Our current methods do not allow us to directly examine the zinc content of heterochromatin versus the euchromatin of a differentiated stem cell, though the development of x-ray fluorescence nanoprobes may soon make this possible. With our current resolution limitations, we analyzed the tightly folded chromosomes of the mitotic stem cell. The striking exclusion of zinc from the condensed chromosomes suggests that heterochromatin would also lack or have a low amount of zinc. This result calls into question the suggested zinc binding potential of histones, at least while involved in DNA packing.

We have considered that the zinc influx may promote loading of zinc finger proteins. There are known zinc finger proteins that play a role in specific differentiation pathways. For example, ATBF1 is a transcription factor containing four homeodomains, and 17 zinc fingers required for neuronal differentiation [36]. On the other hand, zinc finger transcription factors typically act as repressors. Accordingly, over a dozen known zinc finger transcription factors are elevated with pluripotency and are repressed with differentiation [37]–[39]. Therefore, it seems less likely that nuclear influx during differentiation is for the loading of zinc fingers.

Although mostly a cytosolic protein, metallothionein (MT) is a prevalent zinc binding molecule and may act as a metallochaperone for zinc into the nucleus. Literature suggests that cellular zinc and MT levels peak during proliferation and differentiation corresponding with a transition of MT to the nucleus [18]. Nuclear metallothionein has been reported in certain cancer cells [40] and in the G1 to S transition of the cell cycle [41]. Metallothionein is a prime suspect for a zinc chaperone to the nucleus during differentiation. These studies have raised the question: what is the purpose of nuclear MT? Metallothionein not only plays a role in zinc homeostasis but is known to have antioxidant activity [42]. In fact, cytosolic oxidative stress has been shown to induce translocation of MT to the nucleus [43]. Thus, if metallothionein proves to be the nuclear zinc ligand, we must consider the possibility that cellular differentiation causes an intracellular production of reactive oxygen species or is related to extracellular oxidative stress. We continue to explore this exciting possibility.

Finding a protein partner for the zinc influx in the nucleus is more complex than finding a zinc protein that is overexpressed with early differentiation. In fact, the increase in nuclear zinc may not be required for *de novo* protein formation at all, but rather for the activation of existing proteins already present in the nucleus. This would add yet another level of control to the nuclear machinery responsible for differentiation. The development of techniques for evaluation of the binding of metals to individual proteins in biological samples may help us begin to address these questions [44]. Differentiation to this point has been mostly accredited to regulation of protein expression and chromatin remodeling. Now, we must consider import of zinc to activate proteins that maintain a steady state of expression as a potential regulatory step.

Beyond nuclear coordination of the influxed zinc, our results raise questions regarding the role of zinc homeostatic controls in differentiation. Is zinc depletion a hallmark of pluripotency, or is zinc increase a requirement of differentiation? hESC transcriptomes have yet to list zinc importers or exporters. Furthermore, since our data averages zinc concentration over several cells, our results fall short of answering weather the zinc influx is a gradual increase over time or an all-or-nothing response. We will continue to pursue the answers to these questions.

In conclusion, we have clearly demonstrated for the first time that, in the early stages of differentiation, nuclei of hESCs elevate their content of zinc and it is either protein or small molecule bound. Not only does this have implications for zinc's role in developmental biology, it has direct medical consequences as this analysis has elucidated another potential control switch for stem cells in the differentiation process. Given the current challenges with maintaining pluripotency, this research and the questions raised by this work demonstrate the need for continued study into the molecular basis for pluripotency and the role of zinc in hESCs.

## Materials and Methods

### hESC culture

Human embryonic stem cell line H9 (WiCell Research Institute, Madison, WI) was cultured as recommended by the WiCell Research Institute in Dulbecco's Modified Eagle's Medium/Ham's F-12 medium (DMEM/F12) (Invitrogen Corp., Carlsbad, California, 11330–032) with 20% Knockout serum replacement (Invitrogen 10828–028), 1 mM L-glutamine (Sigma, St. Louis, MO, G-8540), 0.1 mM β-mercaptoethanol (Sigma M7522), 0.1 mM nonessential amino acids (Invitrogen 11140–050), and 4 ng/mL of b-FGF (Invitrogen PHG0021) on a mitomycin-C-treated mouse embryonic fibroblast feeder layer.

### Spontaneous hESC differentiation

hESCs (passage 26–34) were cultured as above in 6-well dishes for immunofluorescent studies and on silicon nitride windows (area, 2×2 mm; thickness, 1,000 nm; Silson, Ltd., Blisworth, U.K.) for 7 days. On day 7, cells were fixed with fresh 4% formaldehyde in PBS for 30 min. at room temperature and then washed twice with PBS. In 6-well dishes, pluripotency was assessed with Oct4 immunofluorescence. Cells were blocked overnight with a solution containing 10% goat serum and 0.3% Triton in PBS. Cells were then treated with polyclonal rabbit unconjugated primary antibody to Oct4 (Abcam, Cambridge, UK, ab19857), added at a dilution of 1∶400 in a solution of 2% goat serum in PBS for 3 h at room temperature. Cells were then washed twice with PBS/0.05% Triton and then twice with PBS alone. Alexa Fluor 594 goat anti-rabbit IgG (Invitrogen A11012) at a dilution of 1∶500 was applied in 2% goat serum in PBS at room temperature for 2 h. Cells were washed again as before and then counterstained with Hoechst 33342 for 2 min. Cells were washed with and imaged in PBS. Optical images of the cells were captured on an AxioImager.Z1 microscope (Carl Zeiss, Inc., Thornwood, NY) equipped with a Sensicam QE camera (Cooke Co., Auburn Hills, MI) by using Nomarski differential interference contrast microscopy and epifluorescence microscopy with 40x water immersion objectives and DAPI and Cy3 filter sets. Images were recorded and analyzed using Slidebook 4.1 (Intelligent Imaging, Innovations, Dever, CO).

### Retinoic acid treatment

hESCs (passage 26–34) were again cultured in 6-well dishes and on windows. Retinoic acid (Sigma-Aldrich, R2625) at a final concentration of 10 µM (from 10 mM stock solution in DMSO) was added to culture media from day 2–7 after passaging. Cells were fixed and stained as above.

### Activin A treatment

Cells were differentiated to endoderm as previously described [27], [28]. Briefly, H9 cells were cultured as above and were treated with differentiation media from day 4–7 after passaging. Differentiation media was 2% FBS in RPMI 1640, 10 mM HEPES, 2 mM glutamine with a final concentration of 100 ng/mL activin A (R&D systems Inc., Minneapolis, MN, 338-AC). For control cells, activin A was not included in the differentiation media. Cells were then fixed and stained with anti-Oct4 as above. To characterize activin A treated cultures, cells were tested for Sox17 expression as previously described [28]. Specimens were fixed, blocked with 5% normal donkey serum/0.1% Triton-X/PBS for 30 min at room temperature, and exposed to polyclonal goat anti-human Sox17 (R&D systems, AF1924) at a 1∶500 dilution overnight at 4°C. Specimens were washed 3 times with 0.1% Triton-X/PBS and then treated with Rhodamine Red-X-conjugated donkey anti-goat IgG (Jackson Immunoresearch, 705–295–647) at a 1∶200 dilution for 3 hr at room temperature. Cells were washed and counterstained with Hoechst 33342 prior to microscopy as above.

### FluoZin-3, AM ester staining

On day 7 after passaging, non-fixed, spontaneously differentiating cells, RA treated cells, and activin A treated cells were stained with FluoZin-3,AM (Invitrogen F-24195). Optimal dye incubation conditions were empirically determined to be a final FluoZin-3, AM concentration of 2 µM at room temperature for 30 min. Cells were then washed twice with PBS and then incubated for 30 min with PBS to allow complete de-esterification of intracellular AM esters. Cells were counterstained with Hoechst 33342, formaldehyde fixed and imaged as above with FITC and DAPI filter sets.

### X-ray imaging

Formaldehyde fixed cells on silicon nitride windows were imaged with the scanning x-ray microprobe at beamline 2-ID-E at the Advanced Photon Source (Argonne, IL). Specimens were raster scanned with incident x-rays of 10 keV, monochromatized with a single bounce Si 111 monochromator and focused with a Fresnel zone plate (X-radia, Concord, CA) to a spot size of 0.3×0.5 µm. X-ray fluorescence spectra were collected for 1- to 2- sec dwell times with a three-element UltraLE GE-detector (Canberra, Meridien, CT) or single-element silicon drift detector (Vortex EX, SII Nanotechnology, Northridge, CA). Collected specimen spectra were fit against signal from thin-film standards NBS-1832 and NBS-1833 (National Bureau of Standards, Gaithersburg, MD). Fit spectra were then processed into two-dimensional images of metal content using MAPS software [45].
